# Baclofen in the treatment of alcohol use disorder: tailored doses matter

**DOI:** 10.1093/alcalc/agad090

**Published:** 2024-01-23

**Authors:** Renaud de Beaurepaire, Philippe Jaury

**Affiliations:** Renaud de Beaurepaire, GH Paul-Guiraud, 54 Avenue de La République, 94806 Villejuif, France; Faculté de Médecine, Université Paris Cité, Paris, France

**Keywords:** high-dose, adverse effect, benefice-risk, therapeutic alliance, education of patients

## Abstract

**Aims:**

To address the question of tailored baclofen prescribing in alcohol use disorder (AUD) in relation to dose-dependent efficacy and the potential danger of high doses and to provide suggestions for the use of high doses of baclofen in the treatment of AUD. The context is the approvement in France of baclofen in the treatment of AUD without dose limitation, making French physicians, who usually prescribe baclofen in a tailored manner, often use high or very high doses.

**Methods:**

A narrative review of the results of randomized controlled trials (RCTs) and observational studies that used tailored baclofen prescribing and of the severe adverse effects of baclofen that have been reported in the literature.

**Results:**

The results show that RCTs using tailored doses of baclofen in AUD are not completely demonstrative, though they are encouraging according to certain meta-analyses, while observational studies that used tailored doses constantly show a good effectiveness of baclofen treatment. The results suggest that many severe adverse effects of baclofen could be related to a nonrespect by physicians of prescription rules and appropriate treatment monitoring.

**Conclusions:**

The use of tailored doses shows that the dose required to suppress cravings is highly variable, low or high, depending on each case. Analysis of the circumstances in which severe adverse effects occur suggest that a careful monitoring of baclofen prescribing might prevent a large majority of severe adverse effects. We propose that the education of the patients and the prescription skills, seriousness, and availability of the prescribing physicians are of major importance in the managing of tailored baclofen treatment of AUD.

## Introduction

Alcohol use disorder (AUD) is a major public health problem, affecting 5%–10% of the population in almost all countries and being responsible for the deaths of millions of people worldwide each year (American Psychiatric Association 2013; Grant *et al.* 2015; World Health Organization 2018). Current approved medications for AUD are of limited efficacy (Jonas *et al.* 2014), which could in part explain why only a minority of subjects with AUD seek treatment (Schmidt 2016). Baclofen, a gamma-aminobutyric acid-B (GABA-B) receptor agonist, is a promising treatment for AUD. Baclofen is a drug developed in the early sixties for the treatment of muscular stiffness secondary to central nervous system damage. For more than 30 years, baclofen was almost exclusively used by neurologists, but at the beginning of the 2000s, a study suggested that baclofen might be effective in the treatment of AUD patients (Addolorato *et al.* 2000). This finding came after a series of animal studies showing that baclofen suppresses the behavioral effects of alcohol (Cott *et al.* 1976) and decreases the self-administration of alcohol in alcohol-preferring rats (Daoust *et al.* 1987) (see, for review, Colombo and Gessa 2018; Agabio *et al.* 2016). In addition, it was found that gamma-hydroxybutyrate, another GABA-B agonist, is effective in the treatment of AUD (Gallimberti *et al.* 1992; Caputo *et al.* 2009; van den Brink *et al.* 2018).

The effectiveness of baclofen in the treatment of AUD as shown in 2000 in a small open study (Addolorato *et al.* 2000) was confirmed in 2002 by the same group in a randomized controlled trial (RCT) (Addolorato *et al.* 2002). However, 16 RCTs have been conducted since, showing very discrepant results (Addolorato *et al.* 2002, 2007, 2011; Garbutt *et al.* 2010a, 2010b, 2021; Morley *et al.* 2014, 2018; Krupitsky *et al.* 2015; Leggio *et al.* 2015; Müller *et al.* 2015; Ponizovsky *et al.* 2015; Beraha *et al.* 2016; Hauser *et al.* 2017; Reynaud *et al.* 2017; Rigal *et al.* 2020), and more than 20 years after Addolorato’s pioneering work, the effectiveness of baclofen in AUD remains a matter of controversy (see, for review, de Beaurepaire *et al.* 2019). Several reviews and meta-analyses concluded that there was low interest, or no interest at all, in baclofen in the treatment of AUD (Lesouef *et al.* 2014; Bschor *et al.* 2018; Minozzi *et al.* 2018; Pierce *et al.* 2018). However, one meta-analyses reported significantly higher rates of abstinence after treatment completion in AUD patients treated with baclofen compared to placebo (Rose and Jones 2018). Moreover, a recent Cochrane systematic review and meta-analysis of baclofen treatment in AUD provided high-certainty evidence that both high and low doses were effective in increasing the percentage of abstinent days (Agabio *et al.* 2023). Additionally, a review and meta-analysis of the influence of anxiety symptoms on clinical outcome during baclofen treatment showed that abstinence rates at 12 weeks or longer were significantly correlated with the baseline anxiety levels in baclofen-treated patients (Agabio *et al.* 2021). It is, in fact, acknowledged that there is great variability in the results of baclofen RCTs because of the many factors affecting the outcome in these patients (Leggio *et al.* 2013; Agabio *et al.* 2018; de Beaurepaire *et al.* 2019). Among these factors, the dose allowed (from 30 to 300 mg/day depending on the studies) and titration regimen (fixed doses or tailored titration) are important.

France is to date the only country in the world where baclofen is approved in the treatment of AUD. Many reasons might account for this singularity. One is the fact that the first demonstration of the effectiveness of a high dose of baclofen in the treatment of AUD comes from France. Ameisen (2005), a French physician who suffered from alcohol addiction, published an article reporting how he cured himself with a high dose (270 mg/day) of baclofen. Ameisen wrote that baclofen made him completely “indifferent to alcohol.” Later, in 2008, he published a book, “The end of my addiction,” reporting his cure (Ameisen 2008). The book was a great editorial success and was followed in France by an important movement of support for baclofen. Nonprofit associations were created to promote baclofen for the treatment of AUD, baclofen Internet forums appeared, and cured patients were invited to express themselves on radio and TV. They called on government authorities, demanding recognition of baclofen in the treatment of alcohol dependence. They were also criticized, creating a controversy that was largely relayed by the media. This period has been called as that of “the French craze for baclofen” (Rolland *et al.* 2012a). Consequently, a large number of alcohol-dependent subjects sought baclofen treatment. The estimated number of French patients taking off-label baclofen for alcohol dependence exceeded 200 000 between 2008 and 2013 (Chaignot *et al.* 2015). To respond to this unexpected situation, the French Health and Safety Agency (ANSM) launched in 2014, for 3 years, a “Temporary Recommendation for Use” (TRU), which is a regulatory framework allowing physicians to prescribe baclofen (described below). The ANSM (2018) approved the use of baclofen in the treatment of AUD, with a dose limited to 80 mg/day. Higher doses were not allowed because of their potential dangers. Two associations of patients then challenged, in court, the merits of the prescription limited to 80 mg/day. The court ruled in favor of the associations and stated, on 4 March 2021, that baclofen could be prescribed over 80 mg/day (Tribunal administratif de Cergy-Pontoise 2021). Since then, French physicians have been authorized to prescribe high doses of baclofen (with the recommendation to not exceed 300 mg/day) (ANSM 2021).

From 2009, reports of the French Regional Pharmacovigilance Centers repeatedly alerted the ANSM about the high number of cases of severe adverse effects (SAEs) of baclofen across the country (ANSM 2012, 2013) (these notified SAEs are detailed below). Additionally, in 2018, the ANSM, with other sponsors, conducted a study of the adverse effects of baclofen, which showed that there is an increased risk of hospitalization and death in patients taking doses >80 mg/day (Chaignot *et al.* 2018). French physicians, in daily practice, usually prescribe baclofen in a tailored manner, i.e. using doses adapted primarily to how patients feel about their cravings. This leads to the use of very variable doses, from low to high or very high. The possible widespread use of high doses of baclofen might have consequences requiring discussion. The present article addresses the question of tailored baclofen prescribing in relation to dose-dependent efficacy (low dose vs. high dose) and the possible danger of high doses, as well as its implications for treatment management. The method used is that of a narrative review of studies that have used tailored doses of baclofen and of publications of the ANSM reporting baclofen SAE in the treatment of AUD. There are no established limits between low and high doses, but it is typical to consider that low doses are those ≤80 mg/day (Chaignot *et al.* 2018; de Beaurepaire *et al.* 2019; de Beaurepaire and Rolland 2022).

## Clinical studies using tailored doses of baclofen

### Randomized controlled trials

While the results of RCTs using nontailored doses (fixed doses) of baclofen are highly contradictory, and therefore nondemonstrative, the results of most of the RCTs using tailored doses tend to demonstrate a benefit of baclofen treatment (Agabio *et al.* 2018; de Beaurepaire *et al.* 2019). Four RCTs used tailored doses of baclofen (Müller *et al.* 2015; Beraha *et al.* 2016; Reynaud *et al.* 2017; Rigal *et al.* 2020), always compared to placebo. In the 2015 Müller *et al.* study, baclofen produced a significant decrease in alcohol consumption, while the effects on craving were not different from those of placebo. In this study, the maximum daily dose allowed was 270 mg, and the mean daily dose was 180 mg. The consumption outcome criterion was abstinence (defined as negative subjective report plus negative breathalyzer test as well as a level of carbohydrate-deficient transferrin within the normal range, or, if increased, lower compared to the baseline level). Craving was evaluated by a visual analog scale (VAS). No SAE was reported in the study. In the 2016 Beraha *et al.* study, baclofen showed no effectiveness on alcohol consumption and craving. The maximum daily dose allowed was 150 mg, and the mean daily dose was 96.6 mg. The outcome criteria were those of the German Addiction Society. Craving was evaluated by the Obsessive-Compulsive Drinking Scale (OCDS). One SAE was reported in the study: a hospitalization due to constipation. In the 2017 Reynaud *et al.* study, baclofen demonstrated no effectiveness on alcohol consumption but significantly decreased craving. The maximum daily dose allowed was 180 mg, and the mean daily dose was 153 mg. The outcome criterion was abstinence during 20 consecutive weeks. Craving was evaluated by the OCDS. Twenty SAEs were reported in the baclofen arm of the study: hospitalizations for alcohol detoxification (9 cases), falls (4), suicidal ideation (1), depression (3), and baclofen overdose (3). In the 2020 Rigal *et al.* study, baclofen produced a significant decrease in alcohol consumption and no effect on craving. The maximum daily dose was 300 mg, and the mean daily dose was 180 mg. The outcome criteria were the WHO criteria (high risk for consumption of alcohol ≥40 g/day for men and ≥20 g/day for women, low risk for consumption below these measures [World Health Organization 2018]). Eighty-five SAEs were reported in the baclofen arm of the study: death (seven cases—none related to baclofen treatment) and mania (three); other SAEs were not specified. The results of the three studies in which baclofen was effective (alcohol consumption or craving) were those allowing the highest doses, while the study allowing the lowest dose showed that baclofen was ineffective. Because the mean maximum dose in the three studies in which baclofen was effective decreasing alcohol consumption or craving was above 150 mg, it is possible that the lack of effectiveness of baclofen in the Beraha *et al.* study was related to the insufficient dose allowed. It is also interesting to note that in the two studies in which baclofen reduced alcohol consumption, it had no effect on craving, while in the study in which baclofen reduced craving, it had no effect on alcohol consumption. Given the widely accepted notion in addictology of a direct relation between the craving for a substance and the consumption of that substance, the contradictions between these studies are difficult to explain. This might illustrate the great difficulties involved in setting up reliable RCTs in alcohology possibly because of the many factors affecting the outcome, as mentioned before.

### Observational studies

Increasing baclofen doses gradually until patients feel an absence of craving for alcohol is the tailored method that all long-term observational studies used (de Beaurepaire 2012; Rigal *et al.* 2012; Heydtmann *et al.* 2015; Barrault *et al.* 2017; Pignon *et al.* 2017; Pinot *et al.* 2018). These studies have consistently shown a marked effectiveness of baclofen and found that high doses of baclofen were more often effective than low doses. The Rigal *et al.,* de Beaurepaire, and Pinot *et al.* studies were “real-world” studies of large series of outpatients over long periods of time, in which any dose of baclofen could be prescribed (no upper limit of dose) and in which WHO criteria were used as outcome measures. The number of abstinent or low-risk patients increased from 0% at initiation of baclofen treatment to 58% in the Rigal *et al.* 1-year study, 62% in the de Beaurepaire 2-year study, and 52.1% in the Pinot *et al.* 3-year study. The mean maximum doses prescribed in these studies were, respectively, 145 mg/day (range 30–400 mg/day), 147 mg/day (range 20–330 mg/day), and 211 mg/day (range 40–520 mg/day). Six SAEs were reported in the de Beaurepaire study: confusion (5) and mania (1). Ten SAEs were reported in the Pinot *et al*. study: confusion (3), encephalopathy (1), and death (6—none related to baclofen treatment). There was no SAE reported in the Rigal *et al*. study. In the Pignon *et al.* 1-year study, 41% of the patients had a favorable drinking outcome. Low doses (<90 mg/day) and high doses (>150 mg/day), but not medium doses (90–150 mg/day), were associated with a significant favorable drinking outcome. The French Alcohol Society (2015) criteria (threshold: 200 gr of alcohol consumption per week) were used as outcome measures. Two SAEs were mentioned in the study (suicide attempts, hypomania); the number of patients affected was not available. The Heydtmann *et al.* and Barrault *et al.* studies were conducted with patients mostly having liver cirrhosis comorbidity. In the Heydtmann *et al.* 2-year follow-up study, baclofen was effective in reducing alcohol consumption in 19 out of 56 patients (34%), with doses ranging from 5 to 400 mg/day. The outcome criteria were the WHO criteria. There was no SAE reported. In the Barrault *et al.* 1-year follow-up study, in which 65% of the patients were cirrhotic, mean daily alcohol consumption was significantly reduced with baclofen doses ranging from 30 to 210 mg/day. The outcome criterion was a reduction of more than 50% of initial alcohol intake. Two deaths were reported in the study, which were not related to baclofen treatment. Overall, these observational studies using tailored doses—which, taken together, included 718 patients—show that baclofen treatment consistently reduced alcohol consumption and that the effective dose was highly variable from one patient to another, ranging from 5 to 520 mg/day.

### The “Temporary Recommendation for Use” register

The results of the analysis of the TRU register, in which data were recorded and collected by the ANSM, were recently published (de Beaurepaire and Rolland 2022). In 2014, the ANSM granted a TRU regulating the prescription of baclofen in AUD. A TRU is a regulatory framework allowing physicians to prescribe an off-label medication, with the objective of collecting clinical data and implementing pharmacovigilance supervision of the treatment. In the TRU for baclofen, a tailored prescription of baclofen was recommended, with a maximum daily dose of 300 mg/day. Alcohol consumption (in grams/day [g/day]), craving (measured by a VAS), and adverse events were recorded on an Internet portal at each patient visit. The TRU was granted for a 3-year period, from March 2014 to March 2017. The outcome criteria were the WHO criteria. Data from 6939 patients were collected, and 5550 were suitable for analysis. The results showed that most of the 5550 subjects were treated with a dose >80 mg. A group of 169 patients was individualized; it included patients followed for at least 1 year and still under baclofen treatment at their first visit after day 365. This group of patients was considered as particularly suitable for comparison with observational studies, especially the Rigal *et al.* study in which patients were followed for 1 year with a maximum daily dose of 300 mg/day. In this group of 169 patients, the daily consumption of alcohol dropped from 88.4 g/day at baseline to 22.3 g/day at the first visit after day 365, the craving score for alcohol decreased from 6.8 to 2.7 between the baseline and the endpoint, and the proportion of subjects with null or low-risk consumption, according to WHO criteria, increased by 46.75% (from 28.40% to 75.15%) between the two time points. The mean daily dose of baclofen at the endpoint was 110.3 mg/day—a dose comparable to that of observational studies (129 mg/day in the Rigal *et al.* study and 100 mg/day in the Pinot *et al.* study).

The analysis of the data of the TRU confirmed that doses of baclofen >80 mg/day are very often necessary to reach treatment effectiveness, in accordance with observational studies and RCTs using high doses. Additionally, comparisons between patients treated with doses ≤80 mg/day and those treated with doses >80 mg/day showed that doses >80 mg/day were not associated with an increase in adverse events after adjustment for the follow-up duration. Two hundred and sixty-five SAEs were reported, including eight deaths (the causes were not reported). The other SAEs were not specified. A limitation of the TRU might have been bias in the selection and follow-up of participants, as it is likely that most of the physicians who included subjects in the TRU cohort were interested in the use of baclofen in AUD and therefore were particularly attentive to the management of the treatment and prevention of SAEs (de Beaurepaire and Rolland 2022).

## Safety concerns that might limit the use of high-dose baclofen in alcohol use disorder

Baclofen has many adverse effects, most of them benign but some potentially dangerous. As mentioned before, reports of the French Regional Pharmacovigilance Centers (31 in France) repeatedly alerted the ANSM about cases of SAEs. In a 2012 report (ANSM 2012), 100 cases of adverse events were notified, including many cases of SAEs. According to the Office for Human Research Protections (OHRP 2007), the SAEs were the following: mental confusion (4 cases), behavioral disinhibition or mania (2), delusions and/or hallucinations (3), depression with ideas of suicide (1), voluntary self-intoxication (7), lowering epileptic threshold (5), withdrawal syndrome with confusion and delirium (1), baclofen abuse (1), liver function problems (4), hepatic encephalopathy (1), and cardiovascular problems (4). In a 2013 report (ANSM 2013), 263 cases of adverse events were notified, including the following SAEs: seizures (15 cases), depression (21), mania/hypomania (18), mental confusion (19), psychosis (6), severe crooked swallowing (1), baclofen withdrawal syndrome (10), and baclofen abuse (8). Additionally, reports of a dramatic increase of self-poisonings, including suicide attempts and unintentional overdose, were released (Holla *et al.* 2015; Pelissier *et al.* 2017; Reynoard *et al.* 2020). Baclofen SAEs therefore became a real concern. Based on the existing published literature and on reports of the French Regional Pharmacovigilance Centers, we propose that the most dangerous effects of baclofen are the following: mental confusion and other vigilance disorders that may cause accidents, mania, depression and suicides, psychosis, seizures, and sleep apnea.

On the other hand, it might be worth remembering that baclofen has been prescribed by neurologists since the early seventies, so for ~50 years, often in very high doses (Smith *et al.* 1991), even directly infused into the brain (Ochs *et al.* 1989), widely for children, all over the world, generally associated with pharmacovigilance follow-up, and that reports from neurologists of SAE of baclofen have been extremely scarce. According to the 1993 edition of the Physicians’ Desk Reference: “other than initial mild sedation, baclofen has few side effects” (Physicians’ Desk Reference 1993). According to the NHS website 2022: *“*serious side effects of baclofen are rare and happen in less than 1 in 10,000 people” (NHS, 2022). Before the use of baclofen for the treatment of AUD, reported SAEs were mainly a few cases of overdoses in suicidal patients and rare cases of some pathological conditions (seizures, psychosis) that are reviewed below. Therefore, considering the millions of patients treated with baclofen over the last 50 years in neurology, one may think that baclofen is a very safe medication. At least, baclofen does not seem to be particularly dangerous when neurologists prescribe it for patients with neurological disorders. How did it suddenly become dangerous when prescribed to patients with AUD?

### Vigilance disorders

Vigilance disorders include daily somnolence and mental confusion. Somnolence and fatigue are the most common adverse effects of baclofen (Rigal *et al.* 2015; Rolland *et al.* 2015). Diurnal somnolence is, most of the time, a benign event. In general, subjects do not feel somnolence when they are active but become somnolent when they relax. In some cases, somnolence is intense, associated with sleep attacks. Somnolence can be dangerous when one is driving, potentially causing car accidents, or when one is using dangerous tools or machines. When associated with other adverse effects of baclofen such as dizziness and balance disorders, somnolence might lead to the occurrence of falls and bone fractures.

Baclofen can cause mental confusion, a state characterized by an inability to think and correctly speak, associated with disrupted attention, spatial and temporal disorientation, delirium, and often hallucinations, with abnormal behaviors usually accompanying these features. In baclofen-treated patients, mental confusion is mostly related to errors in treatment management. An error can be a too-sharp increase in doses, often in patients who want to reach too rapidly the anti-craving dose—the indifference dose. It also happens that patients forget to take their baclofen dose and later try to make up for it by taking additional doses. In these patients, the concomitant use of baclofen and alcohol can cause memory problems: they do not remember whether they have taken their baclofen tablets, and they can take them again several times in a semiconfusional state until they reach a state of frank confusion. Most of the accidents that occur during baclofen treatment happen in patients who simultaneously take alcohol and baclofen (and sometimes benzodiazepines). These patients lose control and might have severe behavioral disturbances, leading to emergency room admissions. Mental confusion in baclofen-treated patients can also be favored by renal insufficiency. Circulating baclofen is almost completely eliminated by the kidney, and in the case of renal insufficiency, baclofen accumulates in the blood of patients who progressively develop mental confusion because of baclofen overload. Several cases of unintentional baclofen intoxication producing mental confusion have been reported in patients with kidney insufficiency (Aisen *et al.* 1994; Ijaz *et al.* 2015). In the case of a massive overdose, the clinical picture is that of a coma associated with seizures, severe cardiovascular dysfunction, and respiratory depression (Paulson 1976; Ghose *et al.* 1980; Reichmuth *et al.* 2015; Kintz *et al.* 2015; Franchitto *et al.* 2018). Mental confusion is also a symptom of abrupt baclofen withdrawal. Abrupt baclofen withdrawal can cause delirium, hallucinations, confusion, and seizures, as well as life-threatening effects such as coma, respiratory depression, and cardiovascular dysfunction (Terrence and Fromm 1981; Khanal *et al.* 2022). Patients must be well aware that they must never abruptly stop their baclofen treatment (Agabio *et al.* 2018; de Beaurepaire *et al.* 2019).

### Mood disorders

Baclofen can produce mild depressive states characterized by apathy, lack of motivation, and feelings of dullness and joylessness. However, baclofen can also be associated with severe depressive states (Sommer and Petrides 1992), and cases of suicide have been reported during baclofen treatment of AUD (Fraser *et al.* 1991; Léger *et al.* 2017; Pelissier *et al.* 2017). These cases of suicide raise the question of the responsibility of baclofen or that of the AUD background. It is well established that there is a high rate of suicidal thoughts and completed suicides in AUD patients (Cherpitel *et al.* 2004). Additionally, baclofen might have been the means used to achieve suicide in AUD subjects who had never taken baclofen before (Kintz *et al.* 2015). Nevertheless, a direct relationship between baclofen and suicidal thoughts has been reported (Mishra *et al.* 2021). Symptoms of depression and ideas of suicide must therefore be closely monitored in baclofen-treated AUD patients, more specifically in those with a personal or family history of depression (Agabio *et al.* 2018; de Beaurepaire *et al.* 2019).

Regarding mood elevation, the ability of baclofen to trigger mania has been known for a long time (Wolf *et al.* 1982; Yassa and Iskandar 1988; Stewart 1992; Rivollier and Masson 2016). In many cases, baclofen-induced manic episodes happen in patients with a history of bipolar disorder, and it is possible that baclofen lowers the threshold for triggering mania (Shah and Singla 2017). However, cases of *de novo* mania in patients with no history of bipolar disorder have been reported (Geoffroy *et al.* 2014). Abrupt baclofen withdrawal may also cause mania (Arnold *et al.* 1980). The clinical experience shows that baclofen can trigger slight hypomanic episodes, characterized by a feeling of well-being, a decreased need for sleep, and increased activity, with no significant behavioral excitation or disorganization. These episodes of slight hypomania do not require any special therapeutic measures. In the absence of a history of bipolarity, these slight hypomanic episodes should be considered benign. However, in rare cases, patients enjoy feeling euphoria, which makes them increase the doses, potentially leading to severe baclofen misuse (Pelerin *et al.* 2023).

### Psychosis

Psychotic symptoms are most often the consequence of abrupt baclofen withdrawal (Lees *et al.* 1977; Terrence and Fromm 1981; Leo and Baer 2005; Sanders and Ali 2021) or baclofen overdose (Reichmuth *et al.* 2015; Nahar *et al.* 2017; Phu *et al.* 2023)*.* They are generally associated with mental confusion, but cases of psychosis in otherwise unclouded consciousness during baclofen withdrawal have been reported (Malhotra and Rosenzweig 2009). Cases of delusions and hallucinations in clear consciousness have also been reported out of the context of withdrawal or overdose, in patients taking low or moderate doses (Roy and Wakefield 1986; Chawla and Sagar 2006)*.* Intrathecal baclofen can also produce psychotic symptoms (Castaño *et al.* 2009*;*Sagawa *et al.* 2011)*.* Psychosis may also be caused by an overload related to renal insufficiency (Amanda *et al.* 2022)*.*

### Seizures

Seizures are a rare SAE of baclofen. Most of the time, they occur in the context of baclofen withdrawal (Barker and Grant 1982; Hyser and Drake 1984; Kofler and Arturo 1992) or overdose (Kofler *et al.* 1994). They often present in the form of nonconvulsive status epilepticus, associated with confusion, psychosis, and abnormal behavior (Hyser and Drake 1984). A case of *de novo* seizures, out of the context of withdrawal or overdose, has been reported in a patient with no history of epilepsy (Rolland *et al.* 2012b). Baclofen and alcohol may both alter seizure threshold, and the responsibility of each in the occurrence of seizures in AUD patients is, in general, difficult to determine. Baclofen may have pro- and antiepileptic properties, and its ability to favor seizures in patients with epilepsy remains ambiguous (Dugladze *et al.* 2013). A study has nevertheless shown that intrathecal baclofen increases the incidence of seizures in patients with multiple sclerosis (Schuele *et al.* 2005). The use of baclofen in patients with epilepsy must therefore be considered with caution (Agabio *et al.* 2018; de Beaurepaire *et al.* 2019).

### Sleep-disordered breathing

Besides confusion and seizures, baclofen overdose can produce severe respiratory depression and cardiac abnormalities, such as bradycardia, arrhythmia, hyper- or hypotension, and conduction defects (Kiel *et al.* 2015). Outside of overdose circumstances, baclofen can produce sleep apnea in AUD patients (Olivier *et al.* 2016), and a dramatic increase in cases of sleep apnea has been reported that parallels the rise of baclofen use in the treatment of AUD in France (Revol *et al.* 2021). Baclofen itself does not seem to provoke sleep apnea in healthy subjects (Finnimore *et al.* 1995), but it can severely worsen preexisting sleep apnea and other sleep breathing abnormalities or vulnerabilities (Ayas *et al.* 2001; Straus *et al.* 2021). Baclofen can worsen sleep apnea through both central and obstructive mechanisms. Alcohol can also cause sleep apnea, and it has been shown that even light drinkers have more than twice the risk of severe sleep-disordered breathing than nondrinkers (Choi *et al.* 2016). Given the potential deleterious consequences of chronic sleep apnea (Parish and Somers 2004), baclofen-related apnea is a real concern during baclofen treatment of AUD. Obesity is the strongest risk factor for the development of obstructive sleep apnea (Hamilton and Joosten 2017), so the patient’s weight must also be considered.

In conclusion of this section, some remarks can be made that moderate the statement that baclofen is not dangerous when prescribed by neurologists, but becomes dangerous when it is given to patients with AUD. The first is that high doses of baclofen are mostly prescribed by neurologists in hospital settings, thus limiting the risk of misuse and serious adverse reactions. Second, baclofen and alcohol strongly interact. At a molecular level, both directly alter GABA-B function and indirectly alter the activity of many common receptors and pathways (de Beaurepaire 2018). In clinical practice, both baclofen and alcohol produce sedation, dizziness, unsteady gait, sensory alterations, confusion, impairment in attention and memory, and sleep disorders (American Psychiatric Association 2013; Rigal *et al.* 2015; Rolland *et al.* 2015). It is therefore likely that the danger of baclofen in the treatment of AUD is, in part, the consequence of the cumulative adverse effects of the two substances.

## Are severe adverse effects of baclofen in alcohol use disorder preventable?

A report of pharmacovigilance that the ANSM published in March 2015 noted that most cases of baclofen hazardous use and SAE were related to a nonrespect by physicians of baclofen titration, of the criteria of prescription, and of patients’ history of epilepsy and psychiatric disorders (ANSM 2015). The experience of the authors of the present article is that tailored baclofen prescription in AUD is a constraining undertaking. Basic principles of prescription must be respected, and the education of patients is essential to avoid SAE.

### Some basic principles

The prescription of baclofen must be slow and progressive. The authors of the present article increase the dose by 10 mg every 3 days. The dose is increased until patients feel they have no more craving for alcohol. Patients are fully involved in the management of the treatment. It is not the physician who determines the effective dose, but the patient. Increasing doses might be made difficult by the occurrence of adverse effects. Special care should be taken to distinguish mild side-effects from potentially dangerous ones. Before treatment, there is no way to predict whether the patient will need a low dose or a high dose. The results of the observational studies cited above show that the effective dose may vary from 5 mg/day to >400 mg/day. The effective dose must be maintained for at least 6 months and then slowly decreased. Factors such as the occurrence of very stressful events or the real long-term motivation of patients to stop drinking have an impact on the maintenance of a state of indifference. In general, subjects who do not drink at all during the period of maintaining the effective dose will be able to completely stop baclofen, while those who continue to drink small doses of alcohol, not because of significant cravings but out of habit or because they are bon vivants, are at risk of rapid relapse after cessation of baclofen. This general view of the management of baclofen prescription implies that baclofen treatment requires significant attention and availability from the physician with a close therapeutic alliance with the patient.

### The education of patients

A checkup of patients is necessary before baclofen treatment begins. Patients must be clearly informed about the potential danger of baclofen if they have psychiatric disorders, sleep-disordered breathing, cardiovascular disease, renal insufficiency, or epilepsy. Also, they must be informed that most of the adverse effects of baclofen are potentialized by alcohol. The following points must be thoroughly addressed and discussed with the patients before treatment initiation:

- Baclofen must be increased slowly and regularly. Increasing the baclofen dose too rapidly or withdrawing it abruptly can cause confusion, seizures, cardiovascular dysfunction, and respiratory problems. The increase of baclofen must be particularly cautious in patients with cardiovascular or respiratory diseases, especially if they are older.
- The need for patients to drive a vehicle, especially for long distances, should be assessed. Drowsiness is a cause of accidents, and baclofen-induced drowsiness is worsened by concomitant drinking and the use of sedative medications. Baclofen may also alter vision (blurred vision).
- Sleep-disordered breathing often causes somnolence during the day. Patients should be informed that baclofen, as well as alcohol, can worsen sleep-disordered breathing. A polysomnographic examination is needed if patients experience excessive daytime sleepiness or other symptoms of sleep apnea. Patients with sleep apnea must use breathing devices during baclofen treatment.
- Symptoms of depression can arise at any moment during baclofen treatment, even in patients with no history of depression. Signs of depression are generally mild (loss of pleasure or motivation, tendency to isolate), but sometimes more severe. In case of ideas of suicide, the patient must tell the physician immediately. Depressive symptoms during baclofen treatment usually respond well to antidepressants. Patients with bipolar disorder must be informed that baclofen can trigger mania. The possibility of increasing the dosage of mood stabilizers may be discussed.
- Overdose or abrupt withdrawal can cause psychotic symptoms. In addition, baclofen sometimes produces psychotic-like symptoms when the doses are increased during tailored treatment: visual hallucinations, auditory hallucinations (rarely), and sometimes paranoia. These symptoms disappear when the dosage of baclofen is reduced. Schizophrenia or other psychotic disorders are not contra-indications for baclofen treatment. Baclofen does not worsen psychotic symptoms in well-stabilized antipsychotic-treated patients, apart from cases of hazardous baclofen use.
- Baclofen and alcohol may both alter seizure threshold. Baclofen must therefore be prescribed cautiously in AUD patients with epilepsy. The possibility of increasing the dosage of antiepileptic drugs may be discussed.
- Over 80% of baclofen elimination is renal, so renal insufficiency produces an increase in blood baclofen, which may cause mental confusion, seizures, and respiratory problems. Blood creatinine must always be checked before baclofen initiation.
- Regarding the monitoring of treatment, patients must thoroughly understand the importance of strictly following the protocol: baclofen must be taken at precise hours, very regularly. Irregularities in the dosage and the hours of taking increase the frequency of adverse effects and may cause the treatment to fail. When a difficulty in bearing an adverse event occurs, the increase must be suspended until the adverse event disappears or becomes tolerable. At this point, the increase can be resumed.
- Baclofen should preferably be taken before drinking. Baclofen is a preventive treatment. Because it suppresses ideas about drinking and representations of the act of drinking, it is in generally useless to take baclofen after drinking has started. Taking baclofen while drinking does not suppress ongoing drinking. Rather, it might worsen the drinking problem because alcohol and baclofen potentiate each other to produce unwanted effects, which often accompany a decreased ability to control consumption. Patients who take alcohol and baclofen simultaneously might not remember whether they have taken their treatment and so take it several times in a row, which could lead to overdosage and confusion. As far as possible, patients are advised to take baclofen within an hour of the onset of cravings.

It is, therefore, very important to thoroughly inform the patients about the management of the treatment and the nature of adverse effects, more specifically of those which might be life-threatening. The experience shows that educating patients makes them much more attentive to the occurrence of adverse effects and thus helps limit their consequences. Additionally, the role of peer-counseling in the treatment of alcohol dependence, and of other addictions, has been very important in France through Internet forums. It may be interesting to note here that the common actions of baclofen and alcohol have led to the hypothesis that baclofen could be, in part, a substitution treatment of AUD, in the same way as methadone in opiate addiction treatment (Rolland *et al.* 2013; de Beaurepaire 2018).

## Conclusion

RCTs using tailored doses and observational studies show that tailored doses of baclofen are effective in the treatment of AUD and that high doses are often necessary. To determine the effective dose, it is important to first rely on what patients feel and say. Many SAE are related to a nonrespect by physicians of baclofen treatment monitoring. A careful monitoring of baclofen prescription should be able to prevent most of the SAEs. The most important issue in tailored baclofen treatment of AUD should be the prescription skills, seriousness, and availability of the physician in charge of the patients rather than questions of its effectiveness or dangerousness.

## Author contributions

Renaud de Beaurepaire (Conceptualization, Data curation, Formal analysis, Funding acquisition, Investigation, Methodology, Project administration, Resources, Software, Supervision, Validation, Visualization, Writing—original draft, Writing—review & editing [equal]) and Philippe Jaury (Conceptualization, Data curation, Formal analysis, Funding acquisition, Investigation, Methodology, Project administration, Resources, Software, Supervision, Validation, Visualization, Writing—original draft, Writing—review & editing [equal])

## Conflict of interest

P.J. has received fees from Ethypharm Laboratories. R.B. has no conflicts of interest.

## Funding

No funding.

## Data availability

There are no research data (the article is a review).
